# Cavotricuspid Isthmus Anatomy Determines The Success Of Remote Controlled Magnetic Bidirectional Block: A Comparsion Between Magnetic 8-mm Solid Tip And 3.5-mm Magnetic Irrigated Tip Catheter

**Published:** 2011-07-03

**Authors:** Buelent Koektuerk, Julian KR Chun, Erik Wissner, Boris Schmidt, Sabine Ernst, Feifan Ouyang, Karl-Heinz Kuck

**Affiliations:** Department of Cardiology, Asklepios Klinik St. Georg, Hanseatic Heart Center Hamburg, Germany

**Keywords:** Atrial flutter, cavotricuspid isthmus anatomy, magnetic navigation, remote controlled, catheter ablation

## Abstract

**Background:**

Cavotricuspid isthmus (CTI) anatomy is associated with a great inter-individual variability. The aim of this study was to compare the magnetic 8-mm tip catheter versus the novel 3.5-mm magnetic irrigated tip catheter in achieving bidirectional right atrial isthmus block and to evaluate the impact of the underlying CTI anatomy on success rate.

**Methods:**

A detailed remote controlled 3-dimensional electroanatomic (3D EA) right atrial reconstruction was performed using the magnetic navigation system with special emphasis to the CTI. CTI anatomy was evaluated utilizing the 3D EA map and classified into (A) simple (flat), (B) complex (pouch-like recess or concave shape) or (C) highly complex (pouch-like recess and concave shape). Patients were treated either with the magnetic 8-mm tip catheter (group I) or the open irrigated magnetic 3.5-mm tip catheter (group II). Primary endpoint was defined as acute bidirectional CTI block utilizing exclusively the remote controlled magnetic navigation system. Secondary endpoint was any procedure related complication and procedure time.

**Results:**

In group I (n=10, 10 males, mean age: 65 ± 9 years) the primary endpoint was achieved in 80% (8/10 pts) requiring a median (Q1; Q3) RF application time of 37.1 min (22.8; 71.5) and a median (Q1;Q3) cumulative energy (CE) of 70.68 kJ (kilo Joule). (10.76;40.59). In group II (n=13, 10 males, mean age: 60 ± 7 years) the primary endpoint was achieved in 92 % (12/13) with a median (Q1; Q3) RF application time of 21.9 min (13.0; 27.0; p value=0.036) and a CE of 33.54 KJ (26.59; 49.22; p value=0.015). Variable CTI anatomy was identified for group I (type A: n=5 pts, type B: n=5 pts, type C: n=0 pts) and group for II (type A: n=4 pts, type B: n=7 pts, type C: n=2 pts). In group I magnetic ablation failure was associated with type B CTI anatomy (n=2 pts) and in group II with type C CTI (n=1). No procedure related complications were observed.

**Conclusion:**

Remote controlled catheter ablation of typical atrial flutter using the magnetic navigation system appears to be safe and feasible. CTI anatomy determines remote controlled magnetic ablation success. Use of the magnetic 3.5 mm irrigated tip catheter should be considered in patients with complex CTI anatomy.

## Introduction

The anatomy of cavotricuspid isthmus (CTI) is highly variable and thereby impacts catheter ablation. Complex CTI anatomy is associated with prolonged procedure times and reduced success rates [1-4]. Remote controlled magnetic (RCM) catheter ablation has been established in the treatment of several arrhythmias [5-10]. It has been demonstrated that the introduction of novel magnetic catheters improved RCM accessory pathway ablation [9].

However, the experience of RCM ablation in the setting of typical atrial flutter (AFL) is limited [11]. Recently, two novel magnetic catheters (8-mm solid tip, 3.5-mm open irrigated tip) have been introduced. The aim of this study was to compare the magnetic 8-mm tip catheter (Navistar RMT DS) versus the novel 3.5-mm magnetic irrigated tip catheter (Navistar Thermocool RMT) in achieving bidirectional CTI block and to evaluate the impact of the underlying CTI anatomy on success rate.

## Methods

### Inclusion and exclusion criteria

A total of 23 patients referred for catheter ablation of AFL have been included in this non-randomized retrospective analysis between May 2007 and October 2008. Inclusion criteria compromised 12 lead ECG documentation of typical AFL, absence of left atrial thrombus determined by transesophageal echo and the absence of exclusion criteria for the MNS Niobe (e.g. pacemaker, implanted ICDs, metallic implants or claustrophobia). No preprocedural CTI imaging was performed for patient selection. All patients gave written informed consent prior to the procedure and the study was approved by the Instutional Review Board. Group I patients were treated with the magnetic 8-mm tip catheter whereas group II pts were treated with the magnetic irrigated 3.5-mm tip catheter. Assignment to each group did depend on the availability of the magnetic catheters.

### Concept of the Magnetic Navigation System

The concept of magnetic navigation system (MNS) has been described previously in detail [5,8,10,11]. In brief, the MNS consists of 2 computer-controlled permanent magnets positioned on opposite sides of the patient's table. These magnets create a steerable external magnetic field (0.08 T) steering the distal tip of the magnetic catheter embedded with small magnets. In conjunction with a motor drive system (Cardiodrive, Stereotaxis) complete remote-controlled navigation and ablation is enabled by advancing or retracting the catheter.

### Electrophysiological Study and Mapping

Vital parameters such as arterial blood pressure and oxygen saturation were monitored throughout the entire procedure. All procedures were performed under conscious sedation using boluses of midazolam, fentanyl, and a continuous infusion of propofol (1%). One 6F catheter decapolar catheter (6-F, Parahis, Biosense Webster) was placed within the coronary sinus (CS) and a second along the His bundle region. One 7F multipolar catheter (Halo, Biosense Webster) was placed anterior to the crista terminalis at the right free wall close to the tricuspid annulus (TA). A detailed remote controlled 3-dimensional right atrial reconstruction (CARTO RMT, Biosense Webster) was performed using the MNS in conjunction with the Cardiodrive (Stereotaxis) catheter advancer system with special emphasis to the region of the CTI. The magnetic catheter was placed in a 6 o'clock position at the tricuspid annulus and slowly pulled back by 3mm steps until the IVC was reached. Additional pull back maneuvers were performed septally and laterally to that initial line to further delineate the patient's CTI anatomy. After AFL termination ablation was continued during sinus rhythm until bidirectional block was demonstrated.

### Ablation procedure

Using a Stockert RF generator, ablation was performed in group I (magnetic 8 mm-tip catheter) in a temperature-controlled mode with a target temperature of 55-60ºC and a maximum energy of 40 Watts. Maximal RF application duration was 120 s. In group II (magnetic 3.5-mm tip irrigated tip catheter) the ablation was performed in a temperature-controlled mode with a target temperature of 43ºC and a maximum energy of 30-40 Watts with a flush rate of 17-30ml/min. Maximal RF application duration was 110 s. Ablation parameters were set according to the manufacturer's recommendations (Biosense Webster, USA). If ablation failed, support by a long sheath (St. Jude, SR0) was allowed. If this did not lead to ablation success or total RF application time exceeded >50 min switch to manual 3.5-mm irrigated tip ablation (Navistar Thermocool, Biosense Webster) was indicated.

### Fluoroscopy exposure

Fluoroscopy times from the control room and from the examination room were noted utilizing a custom-made instrument (Siemens, Forchheim, Germany) allowing for quantification of (1) total fluoroscopic exposure and (2) fluoroscopic burden for the patient and the investigator.

### Endpoints

#### Primary endpoint

The primary endpoint was defined as remote controlled magnetic ablation of CTI with bidirectional block.

#### Secondary endpoints

Secondary endpoints were defined as procedure related complications or AFL recurrence during the follow-up time.

### Definitions

#### Ablation duration

Ablation duration was defined as the time [min] to achieve bidirectional CTI block, measured from the first RF application.

#### Radiofrequency current application time

RF application time was defined as the cumulative time of RF delivery to achieve bidirectional CTI block. Time among RF applications, when no energy was applied was not accounted for.

#### Cavotricuspid Isthmus Anatomy Classification

**Figure 1:**
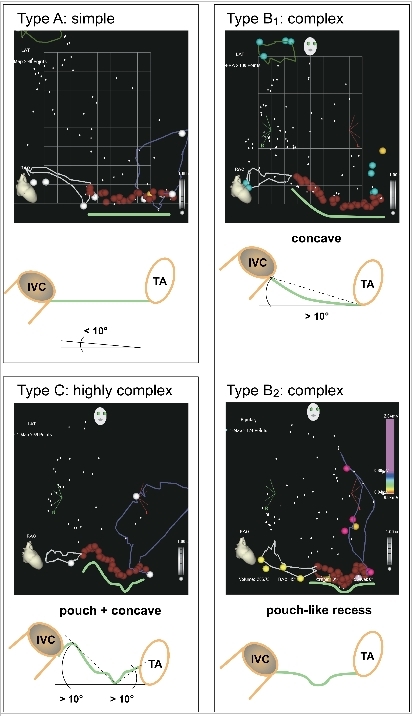
CTI anatomy classification: CTI anatomy was evaluated utilizing the 3D-RA CARTO map (RAO 30º view) and classified into: (A) simple (flat: all CTI points on a straight line between TA and IVC; no angle deviation >10º ), (B) complex (not flat but either pouch-like recess or concave shape; one angle deviation >10º) or (C) highly complex (not flat but pouch-like recess and concave shape; 2 angel deviations >10º).

The CTI length was measured as the shortest linear distance in the mid-inferior aspect between the lower hinge point of the TA and the IVC within the 3D-RA CARTO map. CTI anatomy was evaluated utilizing the 3D-RA CARTO map (RAO view) and classified into: (A) simple (flat: all CTI points were on a straight line between tricuspid annulus and IVC; no angle deviation >10º), (B) complex (not flat but either pouch-like recess or concave shape; one angle deviation >10º) or (C) highly complex (not flat but pouch-like recess and concave shape with at least 2 angle deviations >10º) as shown in Figure 1.

#### Definition of difficult and easy procedures

RCM procedures were classified as "difficult" if bidirectional CTI block could not be achieved or whenever ablation duration exceeded >40 minutes. All remaining procedures were considered as "easy". This is in line with previous published studies where higher-than-median is set as threshold [12].

### Post ablation treatment and follow-up

In all patients pericardial effusion and pneumothorax were ruled out (transthoracic echocardiogram, chest X-ray) after the procedure. If indicated oral anticoagulation was initiated. At day one after the procedure in all patients a 12 lead ECG and a Holter ECG was performed. Follow-up visits were scheduled at month 1, 3 and 6 and included 12-lead ECG, Holter ECG and a transthoracic echocardiography (after 3 months). After 6 months telephonic interviews were performed contacting both the referring physician and the patient.

### Statistical analysis

An exploratory data analysis was performed. The continuous variables were expressed as mean ±SD or as quartiles (median(Q1;Q3) where appropriate, and compared between two groups each with the Mann-Whitney U Test. Categorical data was presented as absolute and relative frequency and compared  with the Fisher's exact test. To calculate the prediction of ablation parameters for acute ablation success univariate logistic regressions were performed. A multivariate regression resulted in a complete correct prediction of all 23 cases. All significances were calculated two-tailed and by the exact method. A probability value <0.05 was considered as statistically significant. All p-values were nominally interpreted and not adjusted for multiple tests. Analysis was performed using SPSS for windows 11.5.2.1, SPSS Inc. 2003.

## Results

### Patients' characteristics

The patients' characteristics are given in Table 1. There were no statistically significant differences between both groups.

### Remote controlled magnetic cavotricuspid isthmus block

The primary endpoint of successful RCM bidirectional CTI block was reached in 87% (20/23 pts): 80% (8/10 pts) in group I vs. 92% (12/13 pts) in group II. All RCM ablation failures 13% (3/20 pts) included the use of a long sheath. In all 3 pts manual irrigated 4-mm tip catheter accomplished CTI block. Ablation failures (n=3 pts) were associated in group I with type B CTI anatomy (n=2 pts) and in group II with type C CTI (n=1).

### Secondary endpoints

#### Complications

In group I and group II discrete catheter tip charring was observed in 60% (6/10) and 46% (6/13), respectively, which was recognized as an impedance rise on the catheters. Besides 3 steam pops in group I without complications, no procedure related complications were observed.

### Follow-up

During a median follow up period of 12.9 months (9.1; 13.5) 91% (21/23) of patients were free of AFL recurrence. In both groups one patient experienced AFL recurrence documented by 24h Holter ECG and 12 lead ECG. In both re-studies recovered conduction accross the ablation line was demonstrated.

### Procedural parameters

Total median procedure time for RCM CTI ablation was 101.2 min. (90.2;121.6) and differed significantly between both groups at 109.4 min (101.7-174.13) in group I vs. 95.0 min (90.0;105.3) in group II (p:0.0487) (Table 2).

Bidirectional CTI block required a significantly longer RF application time in group I as compared to group II ([median (Q1;Q3) : 37.13 min (22.79; 71.45)  vs. 21.92 min (13.01; 27.00), p=0.036]. This was associated also with also significantly increased cumulative RF energy in group I compared to group II [median (Q1;Q3); group I: 70.68 kJ. (40.59 kJ; 107.65 kJ) vs. group II 33.54 kJ (26.59; 49.22), p=0.015]. Median total fluoroscopy exposure for all pts was 5.30 min (4,29; 6,33) including 0.9 min (0,42; 1,36) from the control room resulting in a reduced x-ray exposure for the investigator by 17 %.

### Cavotricuspid Isthmus Anatomy

**Figure 2:**
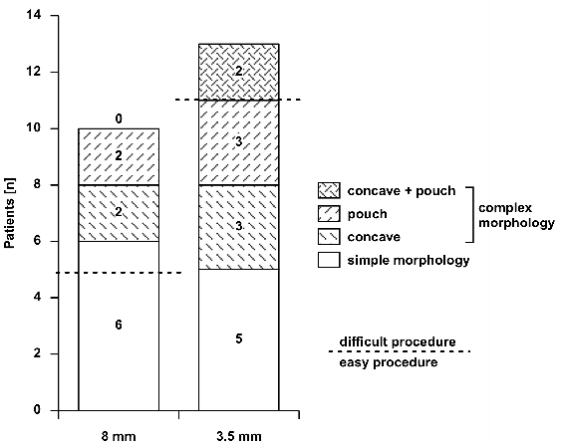
Comparison of rigth atrial anatomy properties of pts in both groups: Distribution of rigth atrial isthmus morphologies in each group (simple, complex and highly complex). Dashed line discriminates between difficult and easy procedures in each group

Total CTI length was 34.0 mm (28.0;44.0).CTI length did not significantly differ between both groups [(p=0.19) but there was a tendency towards a shorter CTI in group I as compared to group II (32.0±10.8 mm vs. 40.9±13.7 mm)].CTI morphology was for group I, type A in n=5 pts, type B in n=5 pts and type C in n=0 pts and for group II, type A in n=4 pts, type B in n=7 pts and type C in n=2 pts. CTI morphology was not significantly different (p=0.41) between both groups (Figure 2).

### Difficult procedures

**Figure 3:**
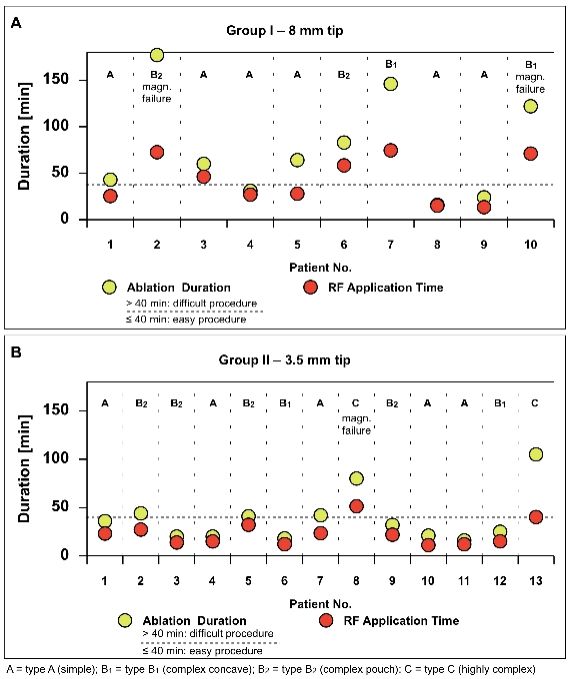
Difficult procedures are signified by longer ablation duration (> 40 min.) depicted as yellow points above the dashed line and a greater difference between ablation duration and RF application time. Upper part of each panel shows the CTI morphology of each patient. As shown in Figure 3a the procedures 2,3,5,6,7 and 10 in group I represent difficult procedures with remarkable differences between the ablation duration depicted by yellow points and RF application time. Patient 2 and 10 are magnetically failed procedures. In group II, case 8 (magnetically failed) and 10 were difficult procedures, reflected by a great difference between the abovementioned parameters as it is shown in Figure 3b

A total of 30% (7/23 pts) of RCM ablation procedures were classified as difficult: 50% (5/10 pts) in group I and 15% (2/13 pts) in group II, including all 3 RCM failures included (type B: n=2, type C: n=1) (Figure 3). In the remaining 4 procedures CTI morphologies were also complex (type B: n=3, type C: n=1). Comparison between easy and difficult procedures in both groups revealed significantly longer median ablation duration [group I: 31.0 min (20.0-41.5) vs 122.0 min (71.5-161.5) (p:0.008); group II:25.5 min (20.0-41.0) vs 92.5 min (80.0;-) (p 0.026)] and median RF application time [group I: 25.4 min (14.3-27.5) vs 71.1 min (52.3-73.5) (p:0.008) ; group II: 15.3 min (12.2-23.5) vs 45.8 min (40.2;-) (p:0.026)].

## Discussion

This study investigated the relationship between CTI anatomy and RCM AFL ablation using two different ablation catheters. As the main finding, it was demonstrated that CTI anatomy determined RCM ablation success for both magnetic ablation catheters. RCM ablation failures or difficult ablation procedures were associated with complex or highly complex CTI morphologies. Interestingly, it appears that the impact of CTI anatomy on ablation parameters utilizing the 8 mm solid tip MC was more pronounced. Comparison of the irrigated and solid tip MC indicated more favourable ablation performance for the novel open irrigation magnetic catheter.

### Impact of CTI anatomy on remote controlled magnetic ablation

Three dimensional EA mapping systems enable a precise reconstruction of the RA and CTI with a good correlation to RA angiography [13-15]. A corresponding classification of the CTI anatomy in simple and complex based on a CARTO-RMT-Map is possible. It has been established that CTI anatomy impacts AFL ablation [1-4,12]. Complex CTI anatomy such as concave shaped CTI, pouch-like recess and/or long CTI were associated with increased RF applications and fluoroscopy time and reduced success rates with manual techniques [1-4,16].

In our series all RCM ablation failures were associated with complex or highly complex CTI anatomy. Use of the 8-mm solid tip catheter was associated with significantly increased biophysical ablation parameters such as RF application time and cumulative energy delivery compared with 3.5-mm irrigated tip catheter. In case of type B CTI (concave shaped or pouch-like recess) or type C CTI (concave shaped and pouch-like recess) the 8-mm electrode may be exposed to decreased blood flow leading to reduced convective cooling of the 8-mm tip catheter with resultant lower resistive heating and power delivery to the tissue [2,3]. In general, the greater electrode-tissue interface with greater resistive heating volume should improve power delivery over the 8-mm tip catheter. However, it is difficult to optimize the electrode-tissue interface for the magnetic 8-mm tip, especially in the context of complex CTI anatomy. In contrast, for the irrigated 3.5-mm tip magnetic catheter local blood flow and the electrode-tissue interface are no essential determinants of resistive heating volume and lesion depth which could be one of the major explanation for the more favourable outcome with less impact of the CTI anatomy on success of the open irrigated 3.5-mm tip magnetic catheter [17,18].

### Ablation performance of magnetic catheters

RF current lesion formation and size is influenced by multiple factors including tissue contact, temperature at the tissue-electrode interface, impedance, power, duration of energy application and catheter orientation. In general, success of a bidirectional CTI block depends on achieving continous transmural lesion creation.

This is the first study to evaluate the ablation performance of both the solid 8-mm tip and the open irrigated 3.5-mm tip magnetic catheter. The data are in line for acute success rates, procedure times and long term success rates of multicenter trials analysing manual AFL ablation catheters [19-21]. However, the overall acute success rate in achieving bidirectional CTI block using the MNS in this study appears to be low compared to manual AFL ablation [22,23]. Recently, contact force has been shown to be a major determinant for ablation lesion size [24]. Reduced contact force exerted by magnetic catheters may have contributed to less power delivery into the tissue and reduced lesion formation as compared to procedures with corresponding manual catheters. This would be in agreement with the recent report of decreased cumulative energy delivery utilizing the electromechanical robotic Sensei™  system (Hansen Medical, USA) to achieve CTI block compared to manual ablation [22].

Interestingly, higher acute success rates for RCM AFL ablation utilizing the magnetic 8-mm solid tip catheter have been reported, which however required high energy settings of up to 70W and a target temperature of 70ºC indicating compensation with power for potentially less contact force [11]. Due to a high incidence of tip charring in both groups in our study even with low energy settings and the risk of steam pops, application of higher energy settings was not done.

In previous studies catheter ablation of typical atrial flutter was associated with a complication rate ranging from 2.6-5.0% [21,23]. Except for minor catheter tip charring in the majority of catheters, the use of RMC was associated with a low complication rate. Furthermore, the magnetic navigation system enables a reduction of the fluoroscopy exposure time for both patient and investigator [19]. Compared to non-magnetic studies this is significantly low and reflects one of the major advantages of the magnetic navigation system for both patients and investigator [24].

## Limitations

This is a retrospective, nonrandomized analysis with a limited number of patients. More prospective, randomized studies including large patients groups are needed, in order to verify the impact of the CTI anatomy on catheter ablation using magnetic navigation system.

In case of ablation failure a cross-over to the catheter of the compared group was not performed. Ablation parameters for both magnetic catheters were set according to the manufactures recommendations. This might be a reason for underpowering especially in group I (magnetic 8-mm solid tip). The incidence of catheter tip charring in both groups and steam pops in group I, however, confirmed not to increase the mentioned energy settings. Analysis of CTI anatomy was performed by 3D EA map only and not assessed by additional imaging modalities such as MRI or CT.

## Conclusion

Remote controlled catheter ablation of typical AFL using the MNS appears to be safe and feasible. CTI anatomy determines remote controlled magnetic ablation success.  Use of the magnetic 3.5 mm irrigated tip catheter should be considered in patients with complex CTI anatomy.

## Figures and Tables

**Table 1:**
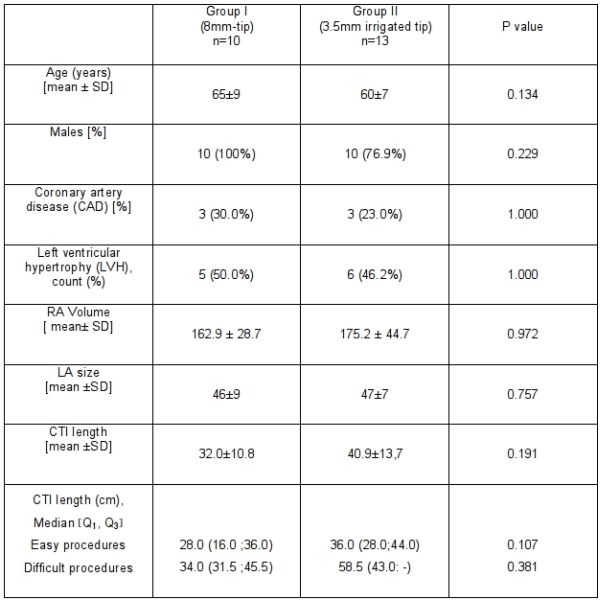
Patients' characteristics: no significant difference with regard to age, gender, EF classification, CAD, LVH , RA volume or LA size

**Table 2:**
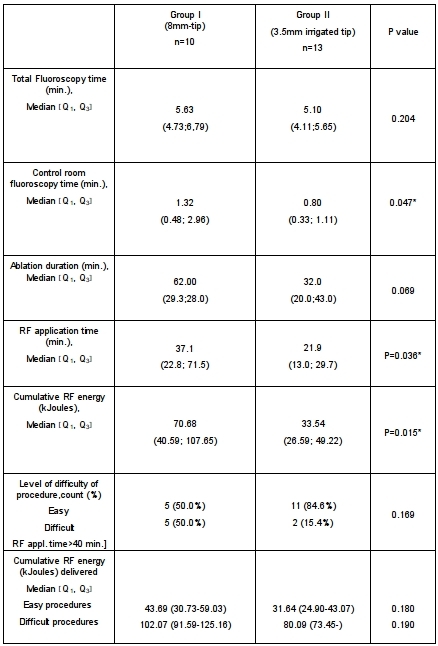
Procedural and ablation parameters for each group [statistical signified different parameters are marked by asterisks]
